# Spatial heterogeneity and predictors of stunting among under five children in Mozambique: a geographically weighted regression

**DOI:** 10.3389/fpubh.2024.1502018

**Published:** 2024-12-18

**Authors:** Tadesse Tarik Tamir, Berhan Tekeba, Enyew Getaneh Mekonen, Alebachew Ferede Zegeye, Deresse Abebe Gebrehana

**Affiliations:** ^1^Department of Pediatrics and Child Health Nursing, School of Nursing, College of Medicine and Health Sciences, University of Gondar, Gondar, Ethiopia; ^2^Department of Surgical Nursing, School of Nursing, College of Medicine and Health Sciences, University of Gondar, Gondar, Ethiopia; ^3^Department of Medical Nursing, School of Nursing, College of Medicine and Health Sciences, University of Gondar, Gondar, Ethiopia; ^4^Department of Internal Medicine, School of Medicine, College of Medicine and Health Sciences, University of Gondar, Gondar, Ethiopia

**Keywords:** children, stunting, predictors, geographically weighted, Mozambique

## Abstract

**Introduction:**

Childhood stunting, a major public health concern in many developing countries, is associated with impaired physical and cognitive development, increased risk of infectious diseases, and long-term adverse health and economic consequences. Mozambique is among the countries with the highest stunting rates in sub-Saharan Africa. This study aims to examine the spatial variation and identify the predictors of stunting among children under the age of five years in Mozambique.

**Methods:**

We utilized children’s recode data and geographic coordinates extracted from the 2022–23 Mozambique Demographic and Health Survey dataset. A stratified two-stage cluster sampling approach was employed. The study included a total weighted sample of 3,910 children under the age of five years. A geographically weighted regression was used to identify predictors of stunting.

**Results:**

The prevalence of stunting among children under the age of five in Mozambique was 31.26% (95% CI: 29.88 to 32.65%). The Nampula (46.00%), Cabo Delgado (43.79%), Manica (40.88%), Zambezia (38.27%), Niassa (35.52%), and Tete (34.85%) regions of Mozambique were identified as hotspot areas of stunting. Several factors were significantly associated with the spatial heterogeneity in stunting, where household wealth index (poor and middle categories) and Mother’s age (15–19 years) were positively associated with higher occurrence of stunting, while having an employed mother and being a child under 2 years of age were negatively associated with stunting occurrence.

**Conclusion:**

The spatial heterogeneity in stunting patterns highlighted by this analysis suggests that a one-size-fits-all approach to address child undernutrition in Mozambique may not be effective. Instead, tailored, location-specific strategies that account for the predictors of stunting are necessary to effectively combat this persistent public health challenge. Policymakers and program implementers should prioritize the hotspot regions for targeted interventions, while also maintaining and strengthening the factors contributing to the lower stunting prevalence in the cold spot areas.

## Introduction

Childhood stunting, defined as a height-for-age z-score less than −2 standard deviations below the median of the World Health Organization (WHO) growth standards, is a major public health concern in many developing countries (1, 2). Stunting is associated with impaired physical and cognitive development, increased risk of infectious diseases, and long-term adverse health and economic consequences (3, 4).

Globally, an estimated 149 million children under the age of five years were stunted in 2020, accounting for 22% of the global under-five population (5). This represents a decrease from 1990, when the global prevalence of stunting was 39% (6). In 2023, the stunting rate among children remained at 24% (7). Projections for 2030 indicate that 23% of children under the age of five will still be stunted, falling short of the 2025 target of 15% (7). Regionally, Africa exhibits the highest rates of stunting (5). The World Health Organization estimates that the current prevalence of stunting among children under five in Africa is approximately 31% (3). Furthermore, a 2024 article highlights that in African countries with low and lower-middle income, the rate is even higher, at 31% (8). While stunting rates have been decreasing over recent decades, the progress has been inconsistent across different regions and countries.

In Mozambique, the prevalence of stunting among children under the age of five has remained alarmingly high, with estimates ranging from 40 to 44% between 2011 and 2019 (9, 10). This places Mozambique among the countries with the highest stunting rates in sub-Saharan Africa. The causes of stunting are multifaceted, stemming from a complex interplay of various factors, including socioeconomic status, Mother’s education, access to healthcare, sanitation, and dietary intake (4, 6).

Several studies have been conducted on stunting in Mozambique (9–15); however, critical gaps remain in the literature. While earlier research has focused on specific geographic areas, a comprehensive nationwide assessment of the spatial distribution of stunting is essential to understand patterns and variations across the country. Additionally, although existing studies have identified various socioeconomic, demographic, and environmental factors associated with stunting (11–15), the underlying drivers of stunting across different regions remain unexamined. Furthermore, the current status of stunting—at both the national and regional levels—has not been thoroughly assessed.

By identifying high-risk areas and key predictors of this regional variation, the proposed study can provide valuable insights for policymakers and public health practitioners to develop targeted, evidence-based strategies to improve the nutritional status of children in Mozambique. Ultimately, this study aligns with national and international priorities in addressing childhood stunting, a key focus of Mozambique’s development agenda and the global Sustainable Development Goals (SDGs). By providing evidence to support effective policy design and implementation, the study can significantly contribute to enhancing child nutrition and health outcomes in Mozambique.

## Methods

### Study setting

**Figure 1:**
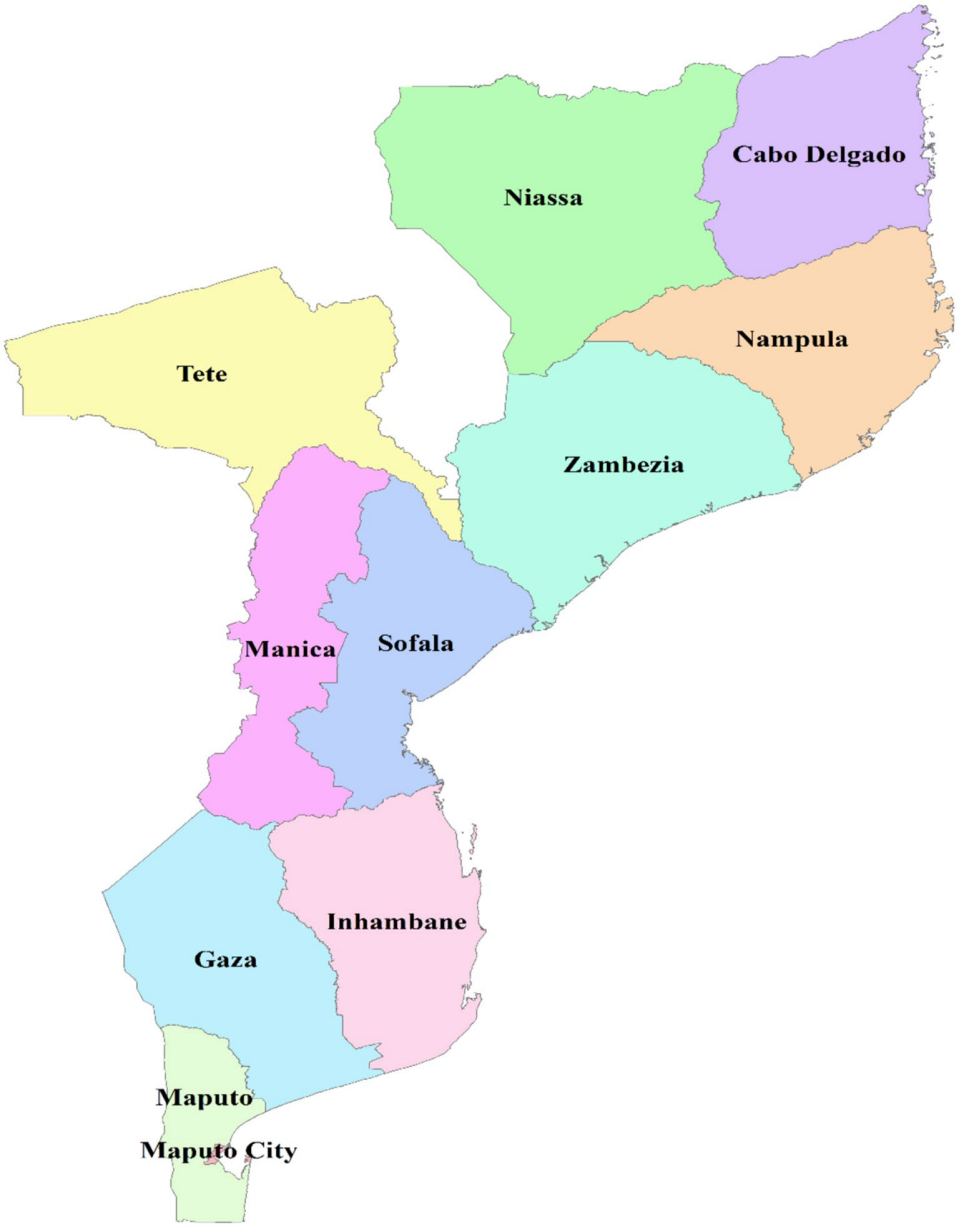
Graphical description of the study setting, Shape file source: Mozambique - Subnational Administrative Boundaries - Humanitarian Data Exchange (humdata.org).

Mozambique is situated on the eastern coast of Southern Africa, opposite Madagascar, and is located between latitudes 10°27’ S and 26°52’ S and longitudes 30°12′ E and 40°51′ E. The country has approximately 2,800 km of shoreline and a total size of around 800,000 km^2^. Administratively, Mozambique is divided into 11 provinces. The 11 provinces of Mozambique are: Cabo Delgado, Gaza, Inhambane, Manica, Maputo City, Maputo, Nampula, Niassa, Sofala, Tete, and Zambezia (Figure 1). Mozambique’s landscape is characterized by a broad plain that is 200–500 meters above sea level and stretches all the way to Beira in the south. The high plateaus of Zimbabwe, Swaziland, Natal, and Transvaal dominate the western regions. The high plateau is situated in the center of the country, along the Zambezi River, between Zambia and Zimbabwe. From Lake Malawi, the country gradually declines toward the Indian Ocean. The current population of Mozambique is projected to be a little over 20 million, with an annual growth rate of 2.5%. Agriculture is the most significant economic sector in Mozambique, providing employment for over 80% of the labor force and serving as the primary means of subsistence for the majority of the population (16).

### Study design and period

The Mozambique Demographic Health Survey data from 2022 to 23 was used to conduct a cross-sectional study.

### Data and sampling

We accessed the publicly available DHS datasets through the DHS Program website at https://www.dhsprogram.com/data/available-datasets.cfm. The analysis was conducted using the children’s recode (KR) data, which was extracted from the Mozambique DHS dataset 2022–23. The standard DHS surveys employ a stratified two-stage cluster sampling design to obtain nationally representative data. First, the country is divided into different strata based on relevant characteristics like urban/rural location or geographic regions. Within each stratum, enumeration areas (clusters) are randomly selected as the primary sampling units. These clusters typically comprise several households. In the second stage, a sample of households is drawn from within each selected enumeration area using either a systematic or random sampling method. Our study population included all children under 5 years of age residing in the sampled households. We excluded children who had visible disabilities affecting their growth or were missing data on key anthropometric indicators necessary for assessing stunting status. In the 2022–23 Mozambique Demographic and Health Survey (DHS), 619 clusters were included. Our study included a total weighted sample of 3,910 children under the age of five years.

### Variables of the study

#### Dependent variable

The dependent variable for this study was the presence or absence of childhood stunting among children under the age of five years. Height or length measurements were available for children aged 0–59 months, which allowed for estimating the proportion of children who were stunted or chronically undernourished. For children aged 24–59 months, standing height was measured, while for children under two years, recumbent length was measured. Stunting was categorized using a binary code: 1 for stunted children and 0 for non-stunted children. Children were considered stunted if their height-for-age measurement fell more than two standard deviations below the WHO Child Growth Standards median (17).

To measure stunting, we utilized the height-for-age z-scores provided in the Demographic and Health Surveys (DHS) data. Specifically, we categorized children as stunted if their height-for-age z-score was below −2 standard deviations from the WHO Child Growth Standards median. In accordance with the guide to DHS statistics, children with missing height values were excluded from both the numerators and denominators. Additionally, children with missing or unknown month or year of birth were flagged and excluded from the analysis of anthropometry indices that rely on age. Furthermore, children with out-of-range or invalid z-scores were also excluded from the numerators and denominators.

#### Independent variables

After an extensive literature review, the study examined several independent variables that may influence the outcomes being assessed. Age is categorized into two groups: birth to 23 months and 24 to 59 months. Gender is classified as male or female. Birth weight is categorized as low, normal, high, or unknown. Mother’s BMI classifies participants into low, normal, or high categories. Mother’s education is divided into no education, primary, secondary, and higher levels. Mother’s age is segmented into three groups: 15–19 years, 20–34 years, and 35–49 years. Mother’s marital status is categorized as not in union or in union.

Father’s education follows a similar structure, with classifications of No education, Primary, Secondary, and Higher. Birth interval is defined as less than two years or two years and above. Family size is categorized as less than five or five and above. Mother’s antenatal care visits are classified as had visits or had no visits. Mother’s anemia is identified as Anemic or not anemic. The wealth index is categorized into three groups: poor, middle, and rich. Media exposure is simply classified as yes or no. The variable Child is twin includes single or multiple classifications. Residence is classified as urban or rural. Lastly, the region includes several categories: Niassa, Cabo Delgado, Nampula, Zambezia, Tete, Manica, Sofala, Inhambane, Gaza, Maputo, and Maputo City.

Birth weight is obtained from reported values according to written records, the mother’s recall, or either source, and is categorized as low (<2,500 g), normal (2500-4000 g), or high (>4,000 g) (18). Body Mass Index (BMI) of mother is categorized as low (<18.5 kg/m^2^), normal (18.5–24.9 kg/m^2^), overweight (25.0–29.9 kg/m^2^), and obese (≥30.0 kg/m^2^) based on a single variable (V445) in the DHS dataset, which is derived from height and weight measurements taken at the time of the interview (18).

The wealth index is a composite measure of a household’s cumulative living standard, calculated by DHS using data on household ownership of selected assets, materials used for housing construction, and access to utilities and services. Households are graded based on the number and type of consumer goods they own, ranging from a television to a bicycle or automobile, as well as housing characteristics such as sources of drinking water, bathroom facilities, and flooring materials. Principal component analysis is used to calculate these scores. National wealth quintiles are determined by assigning the household score to each usual (de jure) household member, ranking each person in the household population based on their score, and then dividing the distribution into five equal groups (poorer, poorest, middle, richer, and rich), each representing 20% of the population (18).

### Data management

The data was processed using STATA/MP version 17.0. This involved editing, confirming, organizing, and recoding the data as appropriate. To extract the proportion of dependent and each independent variable, a cross-tabulation was performed using the variable cluster number (v001), and the results were saved as an Excel CSV file. For the ordinary least squares (OLS) and geographically weighted regression analyses, the relevant variables were loaded into ArcGIS 10.7. This study accounted for the complex survey design of the DHS data used. Specifically, the estimates were weighted using the sampling weights provided in the DHS dataset, and the standard errors of the regression coefficients were adjusted to account for the probability of sample selection and the multistage cluster sampling design. This ensures the estimates are representative of the target population and the statistical inferences are valid.

### Spatial analyses

#### Spatial autocorrelation

The program Arc GIS version 10.7 was utilized to map model parameters between local models and look for geographical variance. The determination of the dispersion, clustering, or random distribution of stunting in Mozambique was achieved by computing the global spatial autocorrelation, also known as Global Moran’s I (19, 20). A geographical statistic called Global Moran’s I uses the complete dataset to generate a single output value between −1 and + 1. This allows for the measurement of spatial autocorrelation. A closer distance from −1 to Moran’s output suggests that the event of interest is scattered, whereas a closer distance from +1 suggests clustering, and a closer distance from 0 suggests a random pattern. A statistically significant Moran’s I (*p* < 0.05) indicates that stunting is not distributed randomly among children under five years old (either dispersed or clustered) (21).

### Cluster and outlier analysis

Anselin Local Moran’s I, which assesses spatial clustering by determining whether a given area exhibits stunting rates significantly higher or lower than its neighbors (22). This statistic helps to identify clusters of similar values and detect outliers in the data. A significant positive value indicates high-high clustering, where areas with high rates of stunting are surrounded by other high-rate areas. Conversely, a significant negative value reflects low-low clustering, where low-rate areas are adjacent to other low-rate areas. Additionally, we observed high-low and low-high clustering, indicating areas where high stunting rates are surrounded by low rates and vice versa (22).

### Hotspot analysis

We conducted hot spot analysis to identify statistically significant clusters of high or low stunting rates. Hot spots are defined as areas where stunting rates are significantly higher than expected based on surrounding populations, while cold spots indicate areas with significantly lower rates (22, 23).

### Spatial interpolation

Based on neighborhood-measured values, the ordinary Kriging method of spatial interpolation was used to forecast the percentage of stunting in un-sampled sites. The Kriging method was preferred above other interpolation methods because an ideal interpolator that provides a minimum mean error (ME) and root mean square error (RMSE) is Kriging interpolation (24).

### Spatial scan statistical analysis

Spatial cluster analysis was conducted using SaTScan version 10.2 software. The Bernoulli model was employed, as the outcome variable (stunting) was binary (yes/no). Coordinates (latitude and longitude) for each survey cluster, along with the case (stunted) and control (not stunted) files, were loaded into the SaTScan program. The maximum spatial cluster size was set to include up to 30% of the total population at risk, in order to capture both small and large clusters of high stunting prevalence. The software then used a likelihood ratio test to identify the most likely (primary) clusters, as well as any secondary clusters that were statistically significant. The likelihood ratio tests determined the clusters that had the highest likelihood of being true clusters, rather than occurring by chance. Statistical significance was set at the *p* < 0.05 level. This spatial scan approach allowed for the detection of geographic areas with a higher than expected prevalence of childhood stunting, which can inform targeted interventions and resource allocation (25).

### Regression analysis

The OLS model was used. The OLS global regression model assumes homogeneity of coefficients for each variable across the study region and estimates the connection between the dependent and independent variables using a single equation (26). The initial stage in selecting the suitable predictor variables for the geographic variation of stunting is the OLS model (27). Verification that there is no stationary percentage of stunting is required before fitting the global and local regression models. Global spatial autocorrelation was used to determine the spatial standard deviation. Global spatial regression modeling was then calibrated to find variables related to the percentage of stunting.

The OLS model is predicated on six assumptions: normality of errors, random sampling, homoscedasticity, no autocorrelation, linearity, and perfect multicollinearity. The variance inflation factor (VIF) values were used to evaluate the multicollinearity. Predictors with VIF values higher than 7.5, or the cut point to indicate that multicollinearity is present, were not seen in this set of data. A global regression model can be expressed as: yi=β0+∑βkxik+εi (28).

Where yi represents the 𝑖^th^ observation of the dependent variable, 𝑥𝑖𝑘 denotes the 𝑖^th^ observation of the kth independent variable, the εi are independent error terms that are normally distributed with a mean of zero, and each 𝛽_𝑘_ must be estimated from a sample of 𝑛 observations.

The local model, Geographically Weighted Regression (GWR), which assumes that the relationship between variables varies spatially (29), was used to model spatially varying relationships after the OLS model’s assumptions were verified and OLS was run. Therefore, the GWR is re-expressed as $yi=\beta 0\left(ui,vi\right)+\sum \beta k\;\left(ui,vi\right)xik+\varepsilon i$ (28).

Where (ui, vi) represents the coordinates of the ith point in space, and βk (ui, vi) is the realization of the continuous function βk (u, v) at point i. This indicates that it can form a continuous surface of parameter values, with the measurements from this surface at specific points reflecting the spatial variability of the surface.

Additionally, between local regression (GWR) and global regression (OLS), AICc and Adjusted R2 were used as model selection criteria. As the best fitted model, the one with the lowest AICc and the highest Adjusted R^2^ was chosen (27).

### Ethical consideration

This study utilized publicly available data from the Demographic and Health Surveys (DHS) Program. Access to the Mozambique 202–23 DHS dataset, including the Global Positioning System (GPS) data, was obtained through the standard DHS data registration and request process. All DHS datasets are de-identified and anonymized to protect the privacy and confidentiality of survey participants. As the data used in this analysis were secondary and anonymized, no additional participant consent was required. The DHS Program has established robust protocols to ensure the ethical collection and use of survey data, in accordance with international standards for the protection of human subjects. This study adhered to these protocols and principles throughout the analysis.

## Results

### Descriptive characteristics of the study subjects

In this study, we analyzed a total weighted sample of 3,910 children under the age of five years. Among them, 777 (41.08%) were male, and 626 (31.00%) were female, both experiencing stunted growth. Notably, 35.61% of children born to mothers with low BMI were also stunted. Furthermore, 41.07% of children born to mothers with anemia faced stunting. Additionally, 179 (50.44%) children born within less than a two-year interval from their preceding birth were stunted. Importantly, 826 (44.20%) children born in households with poor wealth status were found to be stunted. Lastly, 1,112 (40.00%) children living in rural areas exhibited stunted growth (as shown in Table 1).

**Table 1:** Descriptive characteristics of the study subjects.

Variables		Childhood stunting	
		Yes	No
Age	Birth to 23 months	481 (28.51)	1,205 (71.49)
	24 to 59 months	922 (41.45)	1,302 (58.55)
Gender	Male	777 (41.08)	1,114 (58.92)
	Female	626 (31.00)	1,393 (69.00)
Birth weight	Low	74 (40.95)	107 (59.05)
	Normal	389 (29.90)	911 (70.10)
	High	14 (19.37)	60 (80.63)
	Unknown	925 (39.31)	1,428 (60.69)
Mother’s BMI	Low	79 (35.61)	143 (64.39)
	Normal	1,101 (38.05)	1792 (61.95)
	High	219 (35.88)	565 (64.12)
Mother’s education	No education	509 (42.74)	682 (57.26)
	Primary	1,204 (36.97)	706 (63.03)
	Secondary	706 (24.05)	1,204 (75.95)
	Higher	182 (9.82)	574 (90.18)
Mother’s age	15–19 years	149 (38.59)	237 (61.41)
	20–34 years	962 (35.84)	1721 (64.16)
	35–49 years	292 (34.72)	549 (65.28)
Mother’s marital status	Not in union	225 (33.99)	437 (66.01)
	In union	1,177 (36.26)	2,070 (63.74)
Fathers’ education	No education	651 (31.68)	1,404 (68.32)
	Primary	435	752
	Secondary	208 (26.21)	586 (73.79)
	Higher	8 (10.23)	66 (89.77)
Birth interval	Less than two years	179 (50.44)	176 (49.56)
	Two years and above	1,224 (34.42)	2,331 (65.58)
Family size	Less than five	356 (36.82)	611 (63.18)
	Five and above	1,046 (35.56)	1,896 (64.44)
Antenatal care visits	Had visits	594 (31.22)	1,308 (68.78)
	Had no visits	809 (40.27)	1,199 (59.73)
Mother’s anemia	Anemic	915 (41.07)	1,313 (58.93)
	No anemic	487 (28.98)	1,194 (71.02)
Wealth index	Poor	826 (44.20)	1,042 (55.80)
	Middle	318 (59.43)	466 (40.57)
	Rich	258 (20.56)	998 (79.44)
Media exposure	Yes	487 (28.98)	1,194 (71.02)
	No	915 (41.07)	1,313 (58.93)
Child is twin	Single	1,336 (35.19)	2,460 (64.81)
	Multiple	67 (58.62)	47 (41.38)
Residence	Urban	291 (25.71)	840 (74.29)
	Rural	1,112 (40.00)	1,667 (60.00)
Region	Niassa	127 (35.52)	230 (64.48)
	Cabo Delgado	118 (43.79)	152 (56.21)
	Nampula	496 (46.00)	582 (54.00)
	Zambezia	230 (38.27)	371 (61.73)
	Tete	146 (34.85)	272 (65.15)
	Manica	127 (40.88)	184 (59.12)
	Sofala	86 (30.58)	196 (69.42)
	Inhambane	20 (14.91)	116 (85.09)
	Gaza	25 (16.90)	123 (83.10)
	Maputo	18 (8.17)	206 (91.83)
	Maputo City	9 (10.52)	75 (89.48)

### Prevalence of stunting among children under the age of five years in Mozambique

**Figure 2:**
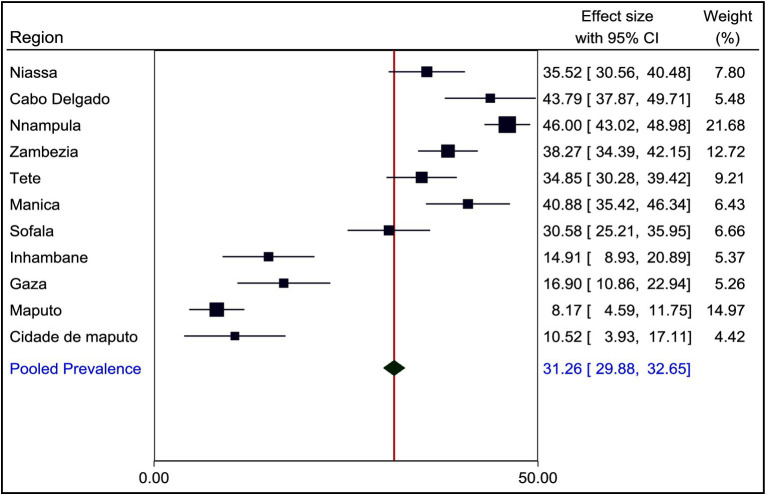
Prevalence of stunting among children under the age of five years in Mozambique, MZDHS 2022–23.

The prevalence of stunting among children under the age of five in Mozambique varies significantly across different regions. Using a forest plot technique, we found that stunting was lower in Maputo (8.17%) with a 95% confidence interval (CI) ranging from 4.59 to 11.75%. In contrast, Nampula had a higher stunting prevalence of 46.00% (95% CI: 43.02 to 48.98%). The pooled estimate for stunting was 31.26% (95% CI: 29.88 to 32.65%) (Figure 2).

### Spatial distribution of stunting among children under the age of five years in Mozambique

**Figure 3:**
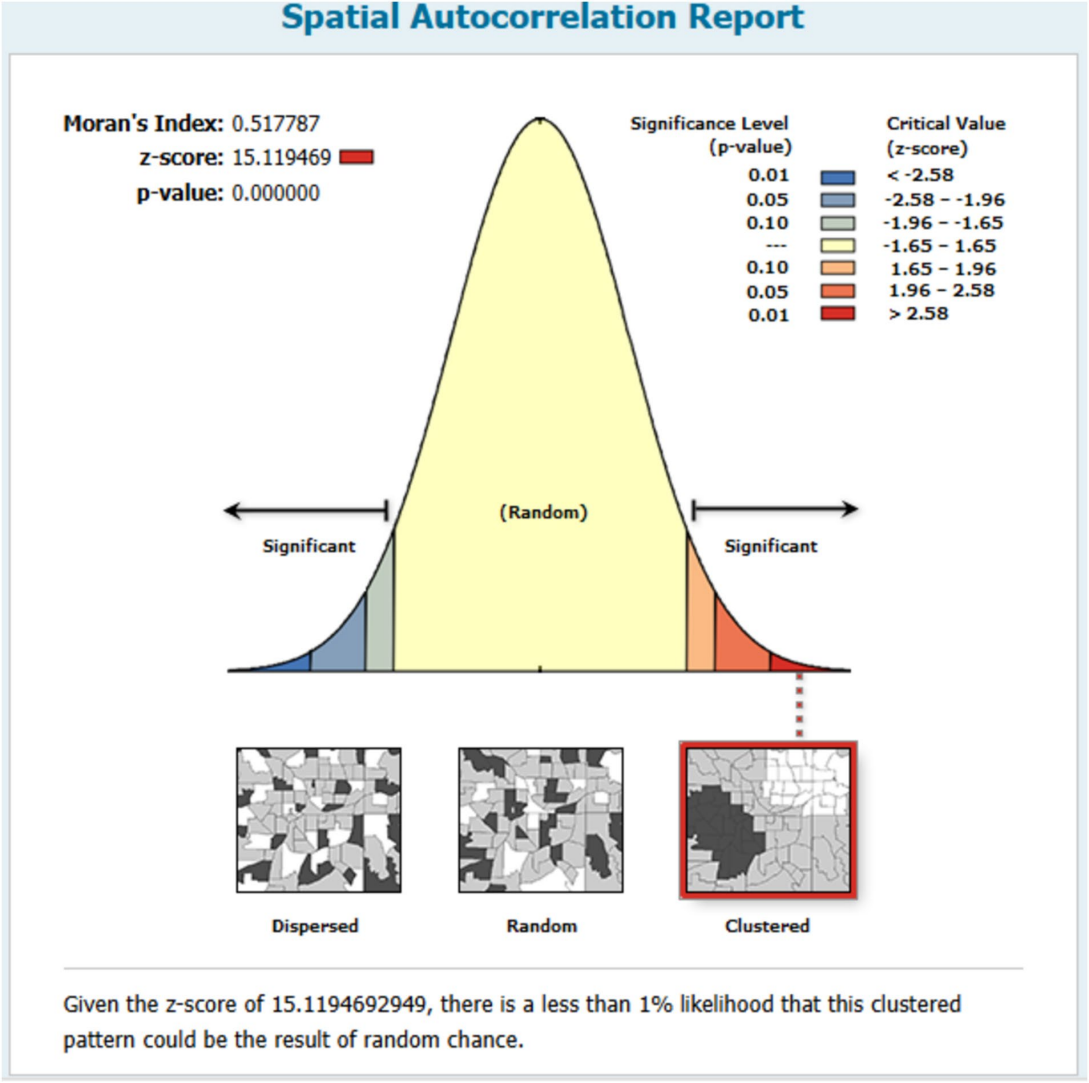
Spatial autocorrelation of stunting among children under the age of five years in Mozambique.

The global Moran’s Index, which measures spatial autocorrelation, revealed significant clustering of childhood stunting in Mozambique. The calculated value of the index was 0.517787, corresponding to a z-score of 15.119469 (*p* < 0.001) (see Figure 3).

### Cluster and outlier analysis

**Figure 4:**
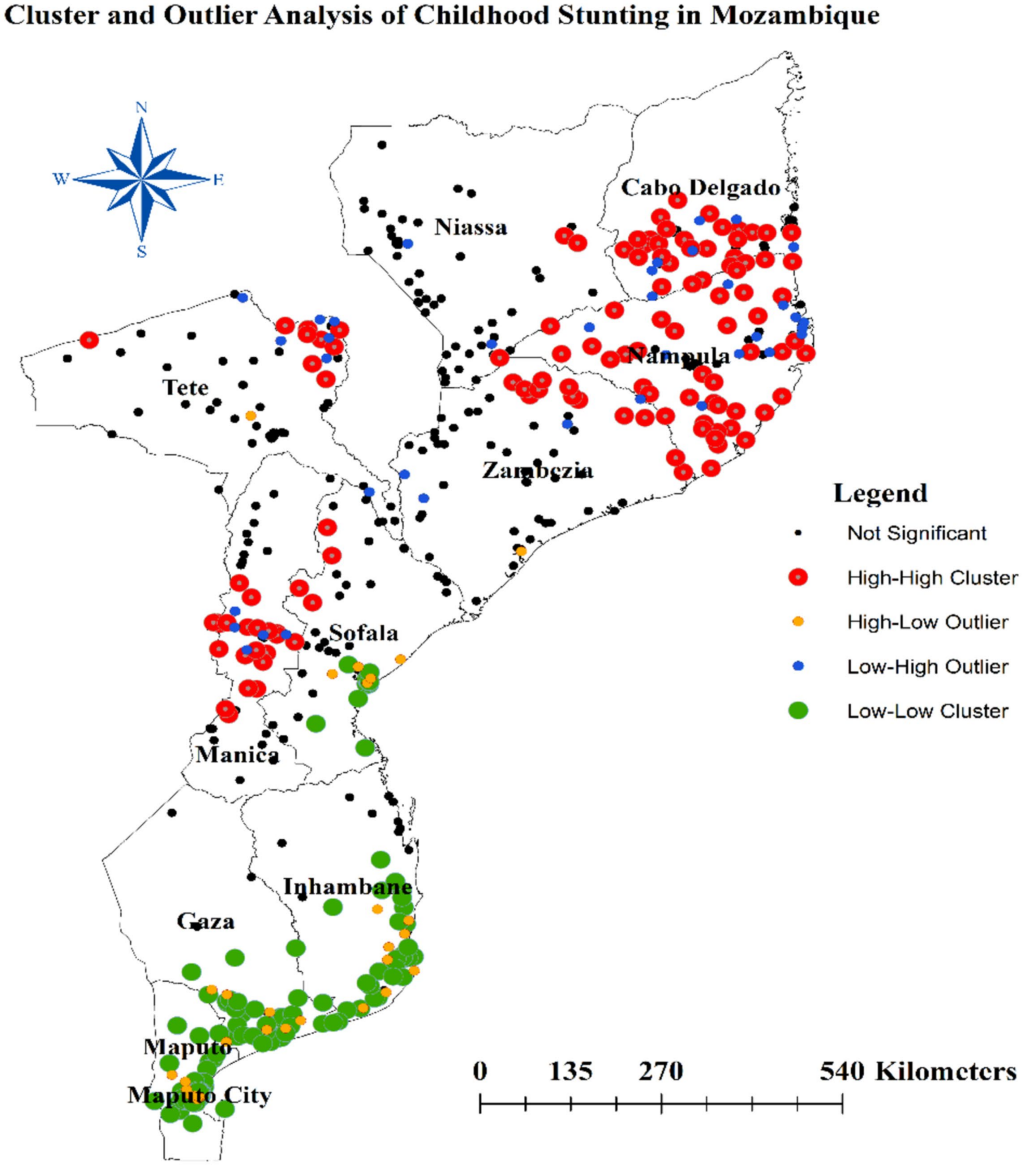
Anselin Local Moran’s I cluster and outlier analysis of stunting in Mozambique.

The Anselin Local Moran’s I cluster and outlier analysis was used to illustrate the geographical distribution of stunting across the country. As shown in Figure 4, this analysis highlights specific clusters and their surrounding areas. In the panels, the red color represents high-high clustering of stunting, indicating areas with elevated rates of similar cases. These specific regions are predominantly found in Cabo Delgado, all areas within Nampula, the northeastern part of Zambezia, the northeast border of Tete, and most of Manica. Conversely, green colors indicate low-low clustering areas, which encompass the southern parts of Inhambane, Gaza, most areas in Maputo, and Maputo city (Figure 4).

### Hotspot analysis of stunting

**Figure 5:**
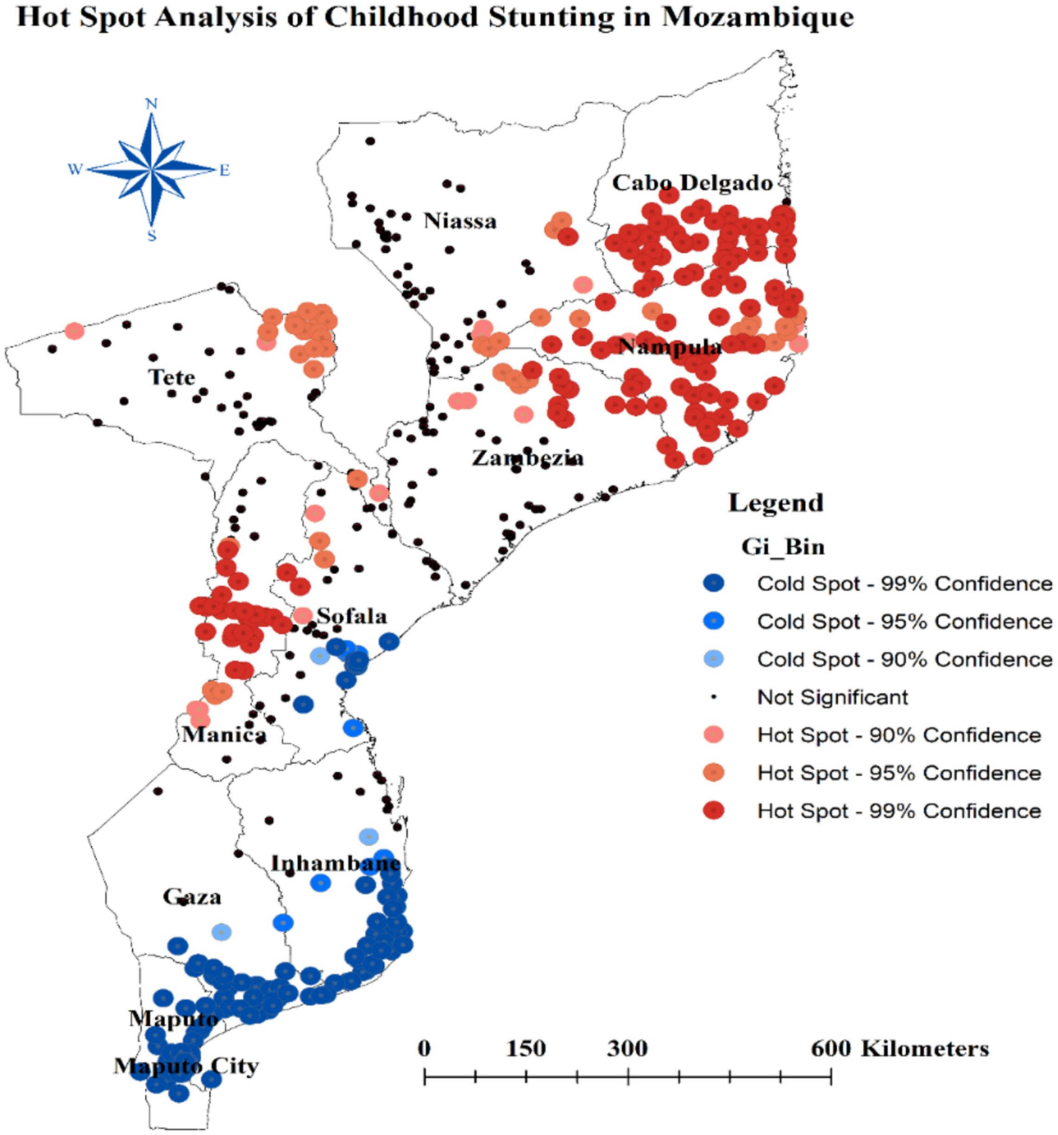
Hot spot analysis of stunting among children under age of five in Mozambique.

The hot spot analysis conducted in this study identified several areas with distinct stunting patterns among children in Mozambique. Hot spot regions, characterized by higher stunting prevalence, include the southeastern border of Niassa, most parts of Cabo Delgado, all areas in Nampula, the northeastern Zambezia, the northeast border of Tete, and most of Manica. Conversely, cold spot areas, where stunting is less prevalent, encompass the southern parts of Sofala, Inhambane, Gaza, most areas in Maputo, and the central parts of Maputo city (Figure 5).

### Spatial scan statistics of stunting

The spatial scan statistics analysis of this study revealed a most likely (primary) and two secondary significant clusters using 30 percent of the total population as the maximum size of the cluster window. The primary cluster, centered at 15.574063°S, 40.417567°E, has a radius of 265.91 km. Within this cluster window, there are 84 clusters (enumeration areas), a population of 1,168, and 548 cases of stunting. The Relative Risk (RR) within this primary cluster is 1.51, indicating that the risk of stunting is 1.51 times higher for individuals living within this 265.91 km radius compared to those outside of it. The *p*-value for this primary cluster is highly significant at less than 0.001, suggesting that the elevated risk is unlikely to have occurred by chance.

The first secondary cluster, centered at 14.916839°S, 39.994143°E, has a radius of 166.58 km. Within this cluster window, there are 68 enumeration areas, a population of 974, and 456 cases of stunting. The RR within this first secondary cluster is 1.46, indicating a 1.46 times higher risk of stunting for individuals living within this 166.58 km radius compared to those outside of it. The *p*-value for this first secondary cluster is also highly significant at less than 0.001. The second secondary cluster, centered at 15.575194°S, 37.611819°E, has a radius of 86.78 km. Within this cluster window, there are 15 enumeration areas, a population of 162, and 88 cases of stunting. The RR within this cluster is 1.55, suggesting a 1.55 times higher risk of stunting for individuals living within this 86.78 km radius compared to those outside of it. The p-value for this second secondary cluster is significant at 0.003 (Table 2).

**Table 2:** Spatial scan statistics analysis of childhood stunting in Mozambique.

Cluster	*N*	Latitude	Longitude	Radius (km)	Population	Cases	RR	LLR	*p*-value
Primary	84	15.574063S	40.417567E	265.91	1,168	548	1.51	44.50	<0.001
Secondary 1	68	14.916839S	39.994143E	166.58	974	456	1.46	33.89	<0.001
Secondary 2	15	15.575194 S	37.611819 E	86.78	162	88	1.55	12.11	0.003

**Figure 6:**
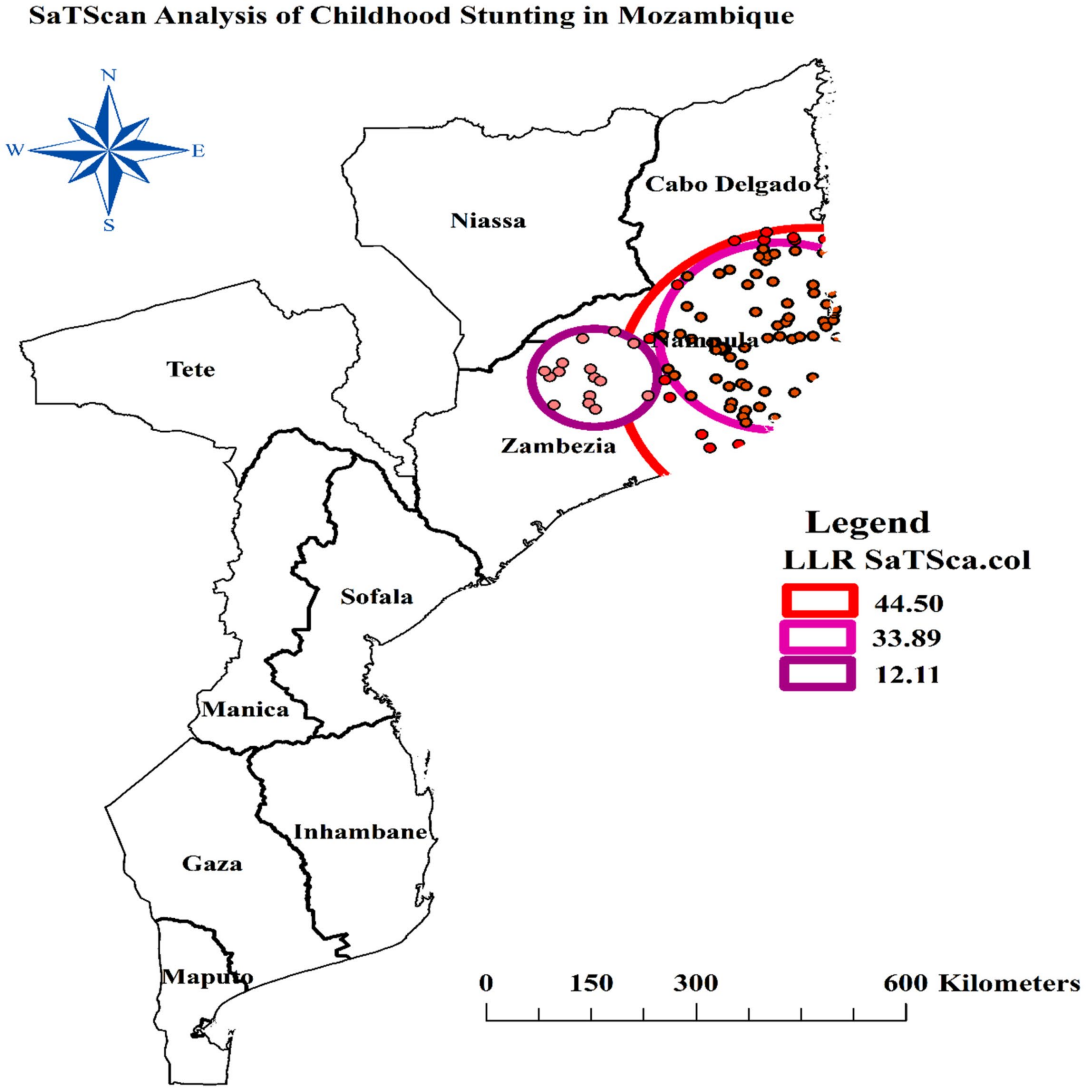
Spatial scan statistics of stunting among children under the age of five in Mozambique.

The most likely cluster windows was observed to cover the south of Cabo Delgado, all of Nampula, and the North and east of Zambezia regions of Mozambique (Figure 6).

### Spatial interpolation of stunting

**Figure 7:**
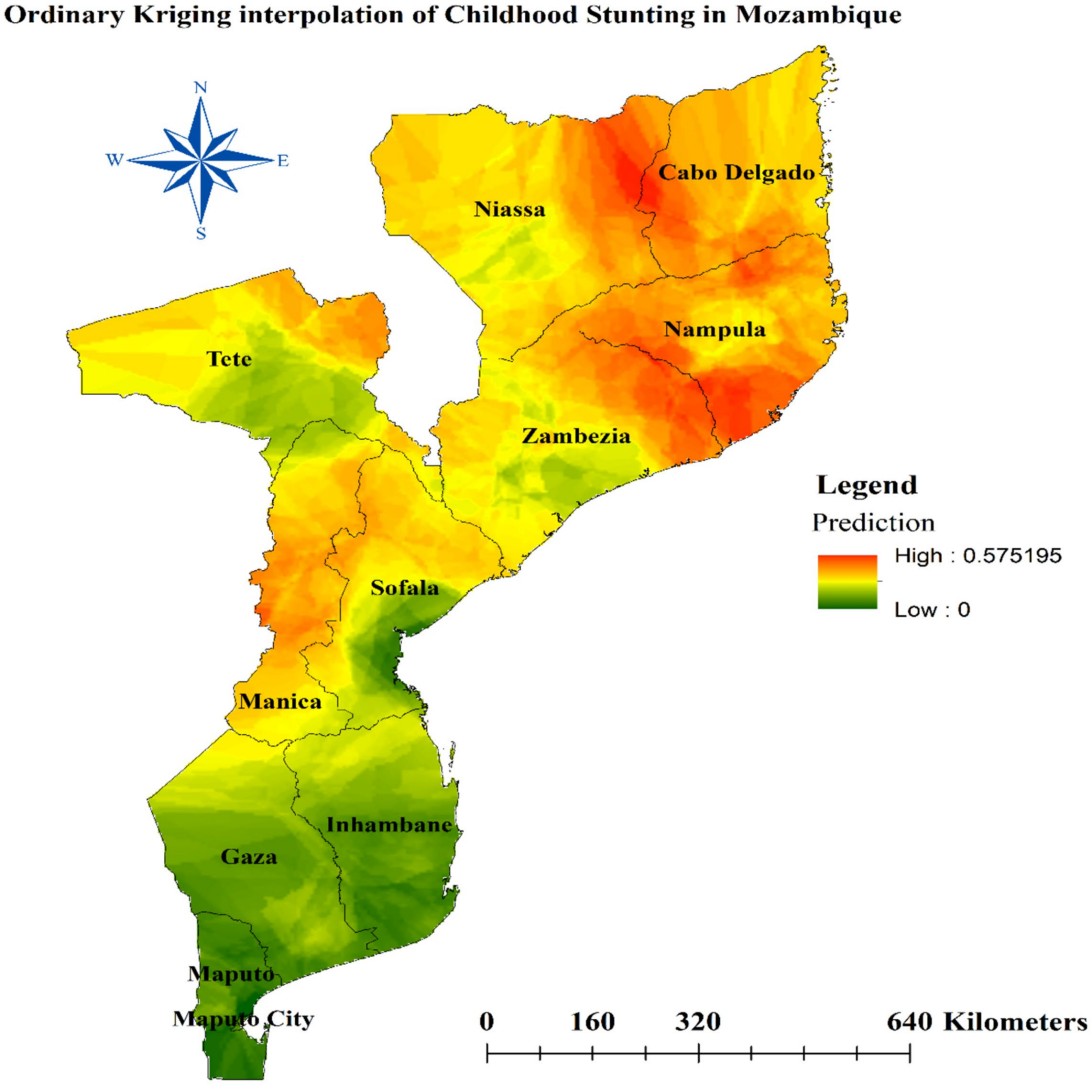
Spatial interpolation of stunting among children under the age of five in Mozambique.

We used a geostatistical technique called ordinary Kriging to map the prevalence of stunting (low height-for-age) among children in different regions of Mozambique. Kriging is an interpolation method that estimates values at unobserved locations based on measurements at nearby locations. The analysis revealed that several regions in northern and central Mozambique had a high proportion of stunted child growth. Specifically, the regions of Cabo Delgado, Nampula, Manica, northern Zambezia, and northeast Tete were identified as areas with elevated levels of childhood stunting, as illustrated in Figure 7 of the study.

### Spatial regression of the predictors stunting in Mozambique

#### Ordinary least squares (OLS) regression

The ordinary least squares (OLS) regression model was used to identify factors associated with spatial variation in childhood stunting. The results indicated that several factors were significantly associated with stunting. The regression coefficients for household wealth index (poor and middle categories) and Mother’s age (15–19 years) were positive, indicating a positive association with stunting. In contrast, the coefficients for Mother’s employment and child age under 2 years were negative, suggesting a protective effect against stunting. Specifically, children from households with a poor or middle wealth index, and children of extremely young mothers (aged 15–19 years) were more likely to be stunted, while children of employed mothers and children under 2 years of age were less likely to be stunted.

Importantly, the analysis did not detect evidence of multicollinearity among the independent variables included in the model. The mean variance inflation factor (VIF) was 4.205, with a minimum of 1.19 and a maximum of 7.22 (see Table 3), which is within the acceptable range for multicollinearity diagnostics.

**Table 3:** Ordinary list square regression summary.

Variables	Coefficients	Robust *t* statistics	Robust probability	VIF
Intercept	0.1450	6.169	<0.001*	------
Male gender	0.002	0.177	0.860	4.66
Multiple birth	0.027	1.186	0.236	1.20
Mother not educated	0.014	1.467	0.143	4.11
Distance to health facility is big problem	−0.009	−1.407	0.160	4.02
Mother employed	−0.023	−2.862	0.004*	1.35
Poor household wealth index	0.024	3.588	0.0004*	7.22
Middle household wealth index	0.027	3.009	0.003*	1.54
Low extreme Mother’s age	0.037	2.465	0.014*	1.42
Upper extreme Mother’s age	−0.019	−1.731	0.084	2.04
Low Mother’s BMI	−0.006	−0.321	0.748	1.79
Mother had no ANC visits	−0.003	−0.321	0.752	6.39
Mather anemic	0.004	0.487	0.626	3.89
Age under two years	−0.026	−2.277	0.023*	5.43
Low birth weight	−0.027	−1.286	0.199	1.19
Five and above family size	0.004	0.805	0.421	3.43
Mother’s marital status (not in union)	0.013	1.238	0.216	1.28
Father not educated	−0.001	−0.153	0.878	2.45
Less than two years birth interval	0.0150	1.001	0.318	1.58
**OLS diagnosis**				
Diagnostic Criteria	Magnitude		*p*-value	
AICc	512.32		---------	
R-Squared	0.3604		---------	
Adjusted R-Squared	0.3342		---------	
Joint F-Statistic	6.133		0.001*	
Joint Wald Statistic	103.564		0.001*	
Koenker (BP) Statistic	68.428		0.01*	
Jarque-Bera Statistic	187.167		0.061	

AICc, akaike information criterion corrected; OLS, ordinary list square; VIF, variance inflation factor.

The adjusted R-squared value of 0.3342 from the global OLS model indicated that 33.42% of the variation in stunting could be explained by the explanatory variables included in our study (Table 3). The significant joint F-statistic and Wald statistic suggested a strong linear relationship between the dependent variable and the independent variables. However, the statistically significant Koenker statistic indicated that the regression model’s consistency varied across the study area, implying that the relationships between variables changed with geographic location. Consequently, a geographically weighted regression (GWR) model would be more appropriate for estimating the model parameters.

### Geographically weighted regression

By comparing diagnostic parameters (AICc and R^2^) for the two models, we observed that AICc decreased from 512.76 (for the OLS model) to 496.99 (for the GWR model). Additionally, the Adjusted R^2^ increased from 0.3342 (33.42%) in the OLS model to 0.3606 (36.06%) in the GWR model. These findings suggest that the diagnostic parameters favor the geographic weighted regression (GWR) model over the ordinary least squares (OLS) model (see Table 4).

**Table 4:** Model comparison of OLS and GWR spatial regression analysis.

Model comparison	OLS	GWR
AICc	512.76	496.99
Adjusted R-Squared	0.3342	0.3606

AICc, akaike information criterion corrected; OLS, ordinary list square; GWR, geographically weighted regression.

**Figure 8:**
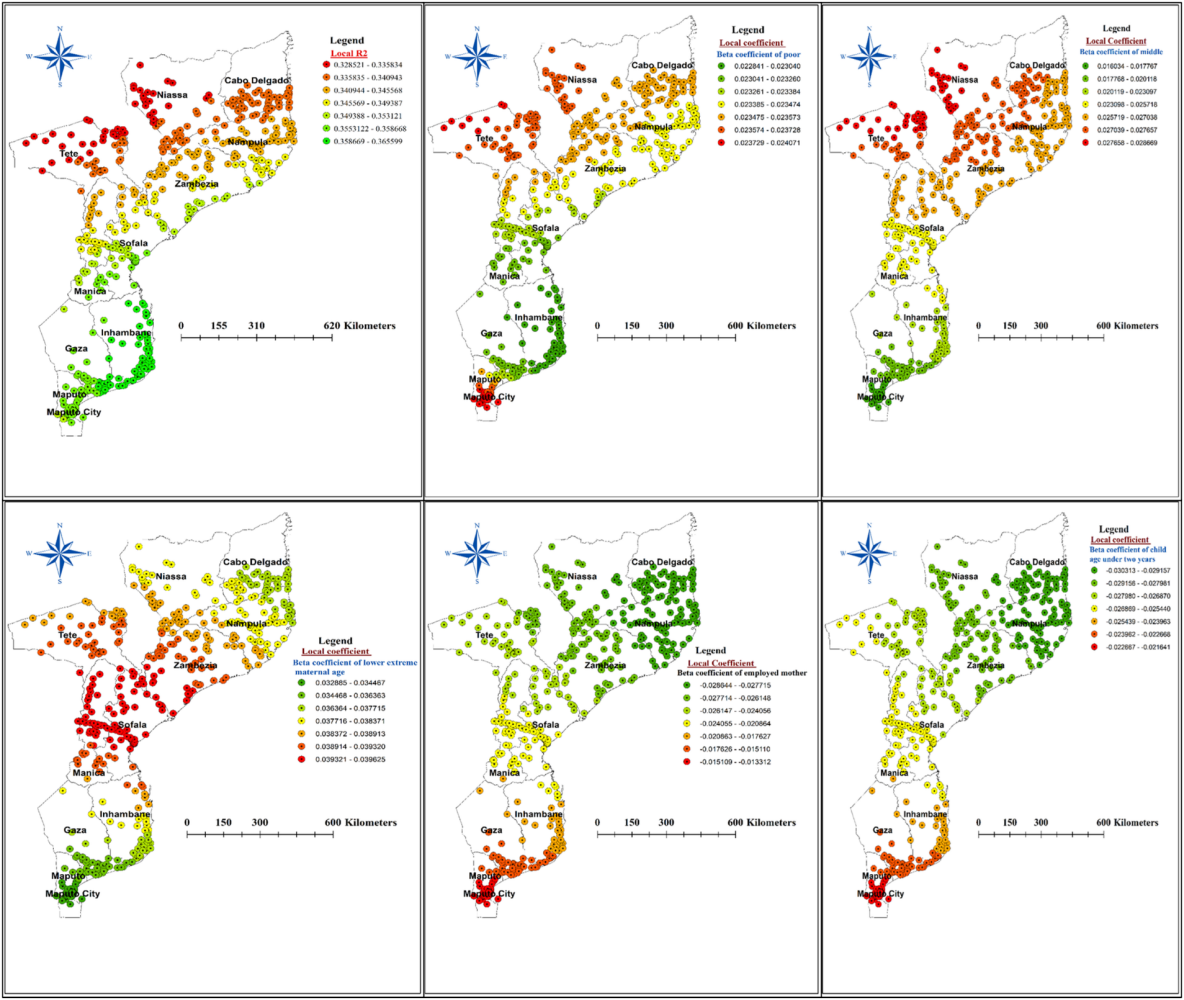
The spatial mapping of local R squared and local regression coefficients of predictors of stunting among children under the age of five years in Mozambique.

Across the study area, the model performance (local R-squared) is depicted in Figure 8. Additionally, Figure 8 displays the spatial distribution of beta coefficients for the five explanatory variables.

The beta coefficient for the poor household wealth index exhibited notable variation across the study area, suggesting an inconsistent relationship between household wealth and the proportion of stunted children. Specifically, a strong positive association (red dotted areas on the map) was observed between the poorest wealth quintiles and childhood stunting in the Tete, Niassa, Cabo Delgado, and Maputo city. This pattern extended to the northern borders of Zambezia, Manica, and Nampula provinces. Conversely, the lowest coefficients for poor household wealth were found in Gaza, Inhambane, Manica, Sofala, and the northern region of Maputo (Figure 8).

Examining the beta coefficient for children from middle household wealth status, we found a concentrated positive association (red dotted areas) in the regions of Tete, Niassa, Cabo Delgado, Sofala, and Zambezia. Additionally, younger mother’s age (15–19 years) had a stronger positive influence on stunting in the Zambezia, Sofala, Manica, and Tete (Figure 8) regions of Mozambique.

Interestingly, being a child of an employed mother and a child under the age of two years were inversely associated with spatial variation in stunting. Specifically, a strong negative effect (green dotted areas on the map) of mother’s employment and child age under two years was identified in Niassa, Cabo Delgado, Sofala, Zambezia and Tete regions (Figure 8).

## Discussion

Stunting, a major public health concern in many low- and middle- income countries, is associated with impaired physical and cognitive development, increased risk of infectious diseases, and long-term adverse health and economic consequences. Mozambique is among the countries with the highest stunting rates in sub-Saharan Africa. This study disclosed prevalence, spatial variation and predictors of stunting among children under the age of five years in Mozambique.

The prevalence of stunting among under-five children in our study was 31.26% (95% CI: 29.88 to 32.65%). This finding is consistent with the challenges faced in the broader sub-Saharan African region, where a recent study reported an overall stunting prevalence of 31.28% (8).

However, the prevalence in our study was higher than the reported stunting rates in neighboring countries such as South Africa (27.4%) (30) and Malawi 26.8% (31). This disparity can be attributed to differences in socioeconomic factors, access to healthcare, and dietary practices between the regions. For instance, South Africa has experienced significant economic growth and improvements in child health indicators in recent years, which may have contributed to its relatively lower stunting prevalence (32). Malawi, on the other hand, has made progress in reducing stunting through various nutrition-specific and nutrition-sensitive interventions, such as the implementation of the National Multi-Sector Nutrition Policy (33).

On the other hand, the prevalence in our study was lower than the reported prevalence in Zambia (34.9%) (34). This difference can be explained by the variations in the underlying determinants of stunting, such as household food security, access to clean water and sanitation, and Mother’s education, which are known to be more favorable in Mozambique compared to Zambia (35).

The findings from the spatial global Moran’s analysis in this study reveal that the proportion of stunting among children under five in Mozambique exhibits significant spatial variation and clustering. This aligns with the results of previous studies conducted in similar contexts. Studies on the spatial distribution of stunting in Ethiopia and Rwanda found notable spatial clustering, with high prevalence of stunting on specific regions of the countries (36–38). Additionally, a review on the determinants of child stunting in Indonesia highlighted the importance of considering spatial factors, as the study noted distinct geographic patterns in the prevalence of stunting across different regions (39). These findings corroborate the spatial clustering of stunting prevalence observed in the current study on Mozambique.

The spatial clustering of stunting can be attributed to various underlying factors that tend to be geographically concentrated. Poverty, poor access to essential services (such as healthcare, sanitation, clean water), suboptimal infant and young child feeding practices, and high disease burden are some of the key determinants of stunting that often exhibit spatial heterogeneity (40, 41). For instance, regions with limited access to nutritious foods and healthcare infrastructure may experience higher stunting rates compared to more resourced areas. Furthermore, environmental factors, such as soil quality, climate, and agricultural productivity, can also contribute to the spatial patterning of stunting. Regions with less favorable environmental conditions for food production and dietary diversity may be more susceptible to higher stunting prevalence (39).

Additionally, the spatial analysis of this study revealed distinct regional patterns in the prevalence of stunting among children under five in Mozambique. The hot spot analysis identified several areas with significantly higher stunting rates, which can be characterized as hot spots. These hot spot regions include the southeastern border of Niassa, most parts of Cabo Delgado, all areas in Nampula, the northeastern Zambezia, the northeast border of Tete, and most of Manica. The high stunting prevalence in these hot spot areas suggests the presence of concentrated risk factors that contribute to poor child growth and development. These may include factors such as limited access to nutritious food (42, 43), poor sanitation and hygiene (44, 45), high disease burden (46, 47), and suboptimal Mother’s and child healthcare practices (48, 49). The clustering of high stunting rates in these regions underscores the need for targeted, area-specific interventions to address the underlying drivers of child undernutrition.

In contrast, the analysis also identified cold spot areas where stunting prevalence was significantly lower. These cold spot regions encompass the southern parts of Sofala, Inhambane, Gaza, most areas in Maputo, and the central parts of Maputo city. The relatively lower stunting rates in these areas may be attributed to better socioeconomic conditions (50, 51), improved access to basic services (52, 53), and more effective nutrition-sensitive and nutrition-specific interventions (54, 55).

The positive and strong relationship between poor household wealth and the proportion of stunted children is evident in provinces of Mozambique such as Tete, Niassa, Cabo Delgado, and Maputo City. This pattern extends to the northern borders of Zambezia, Manica, and Nampula. Additionaly, our examination of the beta coefficient for children from middle household wealth status revealed a concentrated positive relationship with childhood stunting in the Tete, Niassa, Cabo Delgado, Sofala, and Zambezia regions. The observed positive and strong relationship between poor household wealth and the proportion of stunted children in provinces such as Tete, Niassa, Cabo Delgado, and Maputo City can be scientifically justified by several factors. Households with lower wealth status are more likely to have limited access to diverse and nutrient-rich foods, which are essential for proper child growth and development. Inadequate dietary intake, particularly insufficient intake of macronutrients and micronutrients, can lead to stunted growth in children (56). Additionally, poorer households often have limited access to quality healthcare services, including preventive care, routine check-ups, and early diagnosis and treatment of childhood illnesses (57). They may also lack access to clean water, adequate sanitation, and proper hygiene practices, all of which can contribute to the development of stunting through the increased risk of infectious diseases, such as diarrhea and respiratory infections (47).

The finding that the positive relationship between middle household wealth status and childhood stunting is concentrated in the Tete, Niassa, Cabo Delgado, Sofala, and Zambezia regions can be further explained by the uneven development and resource distribution across different regions of Mozambique. Even within the middle wealth range, there may be significant disparities in the distribution of resources and access to essential services, such as healthcare and education, which can contribute to persistent stunting in certain areas (58). Regional differences in cultural practices, dietary preferences, and childcare behaviors can also influence the prevalence of stunting among middle-income households (54). Geographical and environmental factors, such as soil quality, access to clean water, and exposure to environmental toxins, can also affect child growth and development, even in middle-income households, and these variations across different regions may partially explain the concentrated positive relationship between middle household wealth and childhood stunting (58).

Importantly, being a child of an employed mother was inversely associated with spatial variation in stunting. Accordingly, a strong negative effect of mother’s employment on stunting was identified in Niassa, Cabo Delgado, Sofala, Zambezia and Tete regions of Mozambique. This finding that having an employed mother has a strong negative effect on stunting in these specific regions of Mozambique suggests that mother’s employment may be a protective factor against child undernutrition in this context. This aligns with existing research that has found links between mother’s employment and improved child health outcomes, potentially due to increased household income, improved access to resources and healthcare, and changes in intra-household dynamics and decision-making (59–61). However, it is important to note that the relationship between mother’s employment and child stunting can be complex and context-dependent. Factors such as the type of employment, the quality of childcare arrangements, and the distribution of household responsibilities can all play a role in mediating this relationship (62). Additionally, regional differences in socioeconomic, cultural, and environmental factors may contribute to the observed variations in the effect of mother’s employment on stunting across the different regions of Mozambique.

Furthermore, our GWR analysis also revealed a strong negative effect of child age under two years on the prevalence of childhood stunting in the Niassa, Cabo Delgado, Sofala, Zambezia, and Tete regions of Mozambique. This finding suggests that children under the age of two years are less likely to be stunted compared to older children in these regions. This aligns with existing evidence from several studies (63–65). In this respect, it is important to consider that stunting is a long-term condition that develops over a long period of time, and it is hard to reverse once it has happened (3). Consequently, stunting is more common in older children who have experienced malnutrition for a longer period of time than in younger infants who have not (66). In the regions of Niassa, Cabo Delgado, Sofala, Zambezia, and Tete, children under two years of age may have better access to targeted interventions and support services that are focused on this critical period, leading to a lower prevalence of stunting compared to older children (54). Additionally, younger children are more likely to be exclusively breastfed or receive appropriate complementary feeding, which can protect them from the negative consequences of infectious diseases and nutrient deficiencies that contribute to stunting (6).

## Limitations of the study

The findings of this study should be interpreted with consideration of the following limitations. Firstly, the cross-sectional design restricts the ability to draw causal inferences regarding the predictors of stunting, as it does not establish the temporal order of events. Additionally, the study relied on secondary data from the Demographic and Health Survey (DHS) in Mozambique, which may not account for unmeasured confounders, such as genetic factors, that could influence the risk of stunting. Furthermore, the geographical coverage was limited to Mozambique, which may restrict the generalizability of the findings to other countries or regions with differing socioeconomic, environmental, or cultural contexts. Lastly, the measurement of stunting using height-for-age z-scores may be prone to errors or variations in data collection accuracy across different regions or individuals.

### Areas for further research

Further research is needed to evaluate the effectiveness of location-specific interventions aimed at reducing stunting in identified hotspot regions, assessing which strategies yield the best outcomes. Additionally, investigating the role of a mother’s employment on child nutrition and health is crucial, as it is important to explore how different types of employment and work conditions affect stunting rates. Moreover, researching the effectiveness of community engagement strategies can provide insights into promoting nutritional awareness and practices among families, particularly in areas with high stunting rates.

### Policy implications

Policymakers should prioritize targeted interventions in the hotspot regions identified in this study, implementing tailored strategies that address the specific predictors of stunting unique to each area. Resource allocation is also essential; funding programs that support mothers’ employment and education can significantly contribute to lower stunting rates. Furthermore, designing integrated health and nutrition programs that consider the socio-economic context of families will promote comprehensive strategies that address both immediate and underlying causes of stunting. Finally, establishing robust monitoring and evaluation frameworks will allow for the assessment of the impact of implemented policies and interventions, ensuring that data-driven adjustments can be made as needed.

## Conclusion

The spatial heterogeneity in stunting patterns highlighted by this analysis suggests that a one-size-fits-all approach to address child undernutrition in Mozambique may not be effective. Instead, tailored, location-specific strategies that account for the contextual determinants of stunting are necessary to effectively combat this persistent public health challenge. Policymakers and program implementers should prioritize the hot spot regions for targeted interventions, while also maintaining and strengthening the factors contributing to the lower stunting prevalence in the cold spot areas.

## Data Availability

Publicly available datasets were analyzed in this study. This data can be found here: https://www.dhsprogram.com/data/available-datasets.cfm.
